# eHealth as the Next-Generation Perinatal Care: An Overview of the Literature

**DOI:** 10.2196/jmir.9262

**Published:** 2018-06-05

**Authors:** Josephus FM van den Heuvel, T Katrien Groenhof, Jan HW Veerbeek, Wouter W van Solinge, A Titia Lely, Arie Franx, Mireille N Bekker

**Affiliations:** ^1^ Division of Woman and Baby University Medical Center Utrecht Utrecht University Utrecht Netherlands; ^2^ Department of Clinical Chemistry and Hematology University Medical Center Utrecht Utrecht University Utrecht Netherlands

**Keywords:** pregnancy, eHealth, telemedicine, pregnancy complications, fetal monitoring, patient-centered care, pregnancy, high risk, diabetes, gestational, remote consultation, ambulatory monitoring, obstetrics, perinatal care, antenatal care

## Abstract

**Background:**

Unrestricted by time and place, electronic health (eHealth) provides solutions for patient empowerment and value-based health care. Women in the reproductive age are particularly frequent users of internet, social media, and smartphone apps. Therefore, the pregnant patient seems to be a prime candidate for eHealth-supported health care with telemedicine for fetal and maternal conditions.

**Objective:**

This study aims to review the current literature on eHealth developments in pregnancy to assess this new generation of perinatal care.

**Methods:**

We conducted a systematic literature search of studies on eHealth technology in perinatal care in PubMed and EMBASE in June 2017. Studies reporting the use of eHealth during prenatal, perinatal, and postnatal care were included. Given the heterogeneity in study methods, used technologies, and outcome measurements, results were analyzed and presented in a narrative overview of the literature.

**Results:**

The literature search provided 71 studies of interest. These studies were categorized in 6 domains: information and eHealth use, lifestyle (gestational weight gain, exercise, and smoking cessation), gestational diabetes, mental health, low- and middle-income countries, and telemonitoring and teleconsulting. Most studies in gestational diabetes and mental health show that eHealth applications are good alternatives to standard practice. Examples are interactive blood glucose management with remote care using smartphones, telephone screening for postnatal depression, and Web-based cognitive behavioral therapy. Apps and exercise programs show a direction toward less gestational weight gain, increase in step count, and increase in smoking abstinence. Multiple studies describe novel systems to enable home fetal monitoring with cardiotocography and uterine activity. However, only few studies assess outcomes in terms of fetal monitoring safety and efficacy in high-risk pregnancy. Patients and clinicians report good overall satisfaction with new strategies that enable the shift from hospital-centered to patient-centered care.

**Conclusions:**

This review showed that eHealth interventions have a very broad, multilevel field of application focused on perinatal care in all its aspects. Most of the reviewed 71 articles were published after 2013, suggesting this novel type of care is an important topic of clinical and scientific relevance. Despite the promising preliminary results as presented, we accentuate the need for evidence for health outcomes, patient satisfaction, and the impact on costs of the possibilities of eHealth interventions in perinatal care. In general, the combination of increased patient empowerment and home pregnancy care could lead to more satisfaction and efficiency. Despite the challenges of privacy, liability, and costs, eHealth is very likely to disperse globally in the next decade, and it has the potential to deliver a revolution in perinatal care.

## Introduction

### Electronic Health—A New Opportunity?

Health care is facing the emergence of a new range of systems, services, and applications using electronic communication. *Electronic health* (eHealth) is the network of technology applications regarding health issues, including, for example, Web-based informative programs, remote monitoring, teleconsultation, and mobile device–supported care [1]. As the health care costs in developed countries continue to increase, policies for cost reduction without concessions to the quality of care are being imposed. Unrestricted by time and place, eHealth applications also provide solutions for patient empowerment and value-based health care [2]. Patient empowerment is assumed to improve patient participation in medical decision making, commitment to treatment, and thus, health outcomes [3-5]. The boost in patient engagement can be an important factor for the improvement of quality of care and patient safety [6].

**Figure 1:**
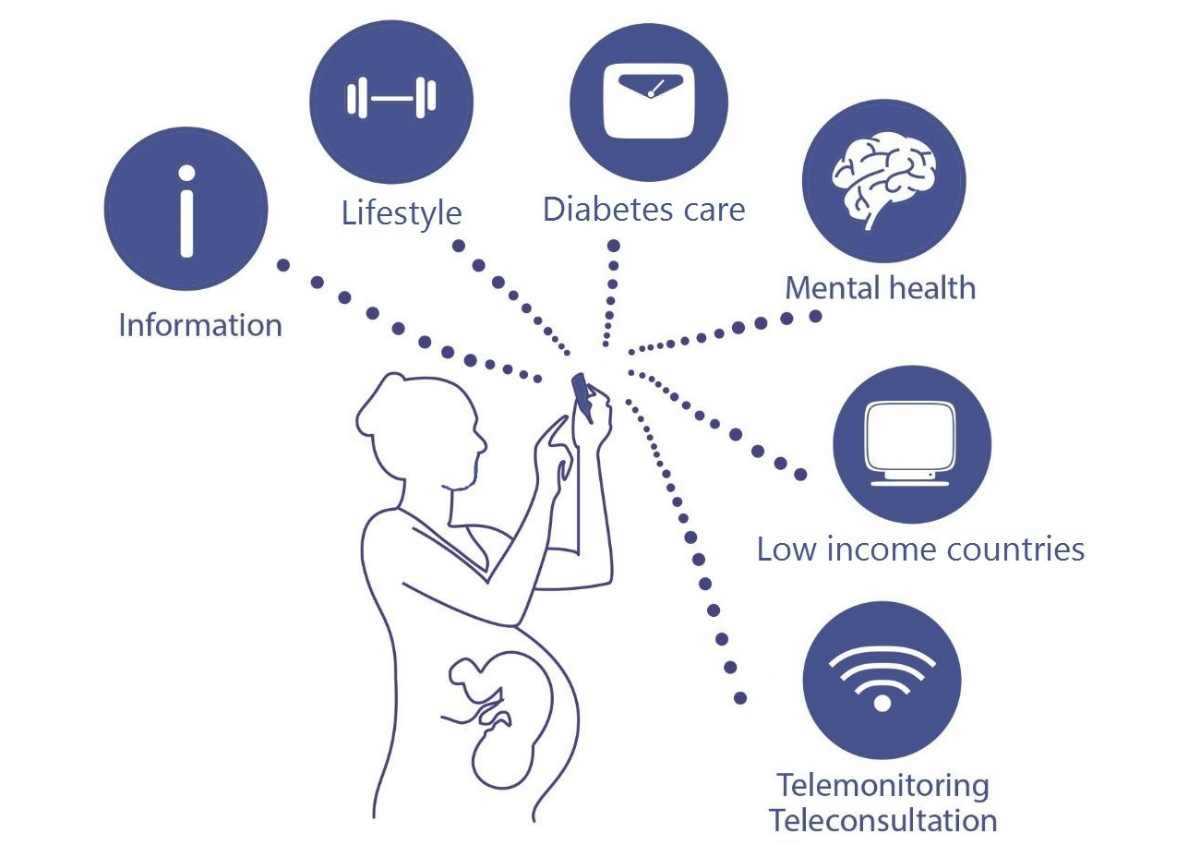
Electronic health (eHealth) solutions in 6 domains of perinatal care.

Young women in their reproductive years are frequent users of internet, social media, and smartphone apps [7]. The internet is ever more utilized for the search of health information on prenatal, perinatal, and postnatal topics [8]. Furthermore, the Web is also used as a forum for the exchange of experiences and peer support [9]. Figure 1 shows multiple domains of perinatal care in which eHealth is already being used by patients and health care providers.

Protocols of professionals’ associations and institutions contain little communication regarding eHealth. No statements are made regarding eHealth in guidelines from the British Royal College of Obstetricians and Gynaecologists, the National Institute for Health and Care Excellence, and the American College of Obstetrics and Gynecology. The Dutch Association of Obstetrics and Gynecology notes that *developments in eHealth should be actively implemented in obstetric healthcare* to *induce the shift of scheduled care to the home setting* and thus lower the in-hospital care burden [10].

### Objective

eHealth has the potential to fulfill a key role in the transformation of the health care system for both patients and caregivers. However, questions are raised if eHealth can deliver the quality of care that is required to remain or even improve health outcomes. It is evident that there is a need for guidance and management of quality standards. Issues of costs and reimbursement; safety of data collection; and storage, privacy, and reliability of information on websites and in apps should also be taken into account.

Our aim is to provide a comprehensive and contemporary overview of the literature on eHealth in perinatal care and assess the applicability, advantages, limitations, and future of this new generation of pregnancy care.

## Methods

A systematic literature search was performed in PubMed and EMBASE in June 2017, combining various synonyms for perinatal care and telemedicine and eHealth (see Multimedia Appendix 1 for the search strategy). Studies reporting the use of eHealth during prenatal, perinatal, and postnatal care were included. Due to the rapid developments in this field and our contemporary scope, we excluded articles describing outdated technologies, for example, fax communication, phonocardiography, and home visits or home care. Screening and reviewing the abstracts and full articles was done by 2 independent authors (JH and KG). Given the heterogeneity in study methods, used technologies, and outcome measurements, results were analyzed and presented in a narrative overview of the literature.

## Results

### Study Selection

Literature search and reference screening provided 71 studies of interest (see Multimedia Appendix 1 for the flow diagram of selection of studies). All articles were categorized in 6 domains, which will be addressed accordingly: information and eHealth use, lifestyle (gestational weight gain, exercise, and smoking cessation), gestational diabetes, mental health, low- and middle-income countries, and telemonitoring/teleconsulting (see also Figure 1). Tables 1-3 show the overview of 71 publications in 6 domains of perinatal care in which eHealth use in patient care was described, implemented, or compared with standard care.

### Information and eHealth Use in Pregnancy

In 15 studies, the characteristics of eHealth users in the perinatal period were described (Table 1). Around 88% (31/35) of participants owned a smartphone [11]. Usage of websites and pregnancy apps for medical information varies from 50% to 98% [7,11-14]. Online information-seeking behavior is common in pregnant women in general and it is not restricted to women with a special profile based on age, education, or social support [7]. Increased knowledge on pregnancy complications has also shown to reduce maternal anxiety and costly hospital visits [15,16]. Factors associated with app use in pregnancy are younger age, nulliparity, lower self-rated health, and higher education. Furthermore, 25.6% (56/219) of questioned women showed interest in a tailored pregnancy app initiated by their health care provider [7,14].

The most searched topics are fetal development, pregnancy complications, healthy lifestyle during pregnancy, generic and specific guidance/advices during pregnancy, and lactation [13,17]. Although they value the Web-based medical information as moderately reliable, 71.3%-75.1% (582/800) of the women do not discuss the information found on internet with their gynecologist [17,18]. One study reported that their lifestyle app helped women to initiate the conversation with their health caregiver on this subject [19].

There is an increasing use of internet for health information, including the perinatal period. However, websites are often contradictory and this may lead to confusion [20]. eHealth may be helpful to address questions through informative websites, apps, and peer support platforms designed by health professionals. Furthermore, eHealth may provide possibilities for decision support in more complicated pregnancies [21].

### Health Outcome After eHealth Intervention

The effect on health is the most important issue to address in the effective implementation of eHealth in perinatal care. Parameters for quality standards include disease outcomes, enhancing patient adherence to treatment, reducing overuse, and increasing access to care [29]. Results of the search showed that most publications focus on the improvement of lifestyle (gestational weight gain, exercise, smoking cessation), gestational diabetes monitoring, mental health, care in lower- and middle-income countries, and telemonitoring.

#### Lifestyle

Our search provided 13 publications describing health outcomes for eHealth interventions on lifestyle during pregnancy (Table 2). Pursuing a healthy lifestyle has proven to be beneficial for pregnancy outcomes such as preterm birth, gestational diabetes, or pre-eclampsia [30-32]. Participant motivation, reducing the dropout rate, and sustainability of long-term results are notoriously difficult in lifestyle studies. Smartphone technologies provide features to overcome these obstacles. Results from feasibility studies show good acceptability, adherence, and engagement for eHealth interventions for healthy gestational weight gain and physical activity, favoring an app over a website [33,34]. Physical activity trials with tailored text messaging (short message service, SMS) services resulted in an increase in step count up to 4 times more than in the control group. In addition, eHealth interventions resulted in better perceived health in pregnancy and lower, healthier gestational weight gain in both nonobese (7.8 kg vs 9.7 kg) and obese women (6.65 kg vs 9.74 kg) [35-37]. Dietary apps directed at healthy gestational weight gain are still in developmental and experimental phase [27,38,39].

Smoking during pregnancy increases the risk of unfavorable pregnancy outcomes. In 2010, approximately 10% of the women smoked cigarettes during pregnancy, especially younger, non-white mothers of a lower social economic status [40,41]. The 2016 review by Heminger et al summarizes the studies performed on SMS programs and mobile apps for smoking cessation in pregnancy [42]. Women participating in SMS cessation programs report relatively high abstinence of 38% in the first week and 54% in the second week (n=20). Biochemically confirmed abstinence rates were 12.5% in participants compared with 7.8% in controls (n=207). Smartphone apps were preferred over SMS-driven programs, as seen in over 10,000 installations of apps compared with 20-800 registrations in SMS programs.

#### Gestational Diabetes

About 5% to 7% of all pregnancies are complicated by gestational diabetes mellitus (GDM) in the United Kingdom and United States (range 1%-25%) [43]. Pregnancies with GDM are associated with perinatal complications such as caesarean section, shoulder dystocia, and neonatal hypoglycemia. Extensive glucose monitoring during pregnancy is a burden for both patients and health care budgets. eHealth in GDM care has evolved most notably of all perinatal appliances of eHealth the last 3 years [44]. We found 13 studies on this topic, including 2 systematic reviews (Table 2). Developments involve smartphone-facilitated remote blood glucose monitoring, management of medication schedules through Web-based or SMS-facilitated feedback systems, and telephone review service to support and supervise glycemic control [45-51]. Overall, studies showed a decrease in planned and unplanned visits by 50% to 66%, whereas no unfavorable differences in glycemic control, maternal, and neonatal outcomes occurred [47-49,52]. Two recent systematic reviews with meta-analysis confirm these results [53,54]. No cost-effectiveness analysis was performed due to insufficient data. There is also increasing evidence of GDM as a risk factor for type 2 diabetes later in life [55]. eHealth programs for follow-up of women with a history of GDM are being developed but need to be examined more thoroughly [45].

**Table 1:** Information and electronic health (eHealth) use in pregnancy: overview of the literature.

Reference	Methods	N	Technology/eHealth intervention
Sayakhot et al [12]	Systematic review (with 7 cross-sectional studies)	3359	Patients’ use of internet for pregnancy information
Ledford et al [22]	RCT^a^ pilot	150	App for pregnancy education and record keeping
Walker et al [15]	Prospective cohort	8	Website for education on placental complications
Bush et al [23]	Before-after study	85	Prenatal care app use and user engagement
Wallwiener et al [7]	Cross sectional	220	Surveys and questionnaires on use of eHealth (smartphones, internet, apps) during pregnancy
Scaioli et al [13]	Cross sectional	1347	Surveys and questionnaires on use of eHealth (smartphones, internet, apps) during pregnancy
Peragallo et al [24]	Cross sectional	100	Surveys and questionnaires on use of eHealth (smartphones, internet, apps) during pregnancy
Lee et al [14]	Cross sectional	193	Surveys and questionnaires on use of eHealth (smartphones, internet, apps) during pregnancy
Lupton et al [25]	Cross sectional	410	Surveys and questionnaires on use of eHealth (smartphones, internet, apps) during pregnancy
Narasimhulu et al [17]	Cross sectional	586	Surveys and questionnaires on use of eHealth (smartphones, internet, apps) during pregnancy
Goetz et al [26]	Qualitative research	30	Focus groups and interviews on eHealth use and implementation (in pregnant women, men, and clinicians)
Willcox et al [27]	Qualitative research	27	Focus groups and interviews on eHealth use and implementation (in pregnant women, men, and clinicians)
Rodger et al [11]	Qualitative research	35	Focus groups and interviews on eHealth use and implementation (in pregnant women, men, and clinicians)
Mackert et al [28]	Qualitative research	32	Focus groups and interviews on eHealth use and implementation (in pregnant women, men, and clinicians)
Lupton et al [25]	Qualitative research	36	Focus groups and interviews on eHealth use and implementation (in pregnant women, men, and clinicians)

^a^RCT: randomized controlled trial.

**Table 2:** Health outcome of electronic health (eHealth) use in lifestyle and gestational diabetes mellitus management in pregnancy: overview of the literature.

Study domain and reference		Methods	N	Technology/eHealth intervention
**Lifestyle: Gestational weight gain, exercise, smoking cessation (13 studies)**				
	O’Brien et al [79]	Systematic review (with 7 studies)	33	Technology-supported diet and lifestyle interventions
	Pollak et al [80]	RCT^a^	33	SMS^b^ programs on healthy lifestyle
	Soltani et al [35]	RCT	14	SMS for heathy lifestyle in women with BMI^c^ >30
	Graham et al [81]	RCT	1335	Internet-based platform to prevent excessive weight gain
	Hayman et al [34]	RCT	77	Web-based physical activity intervention
	Huberty et al [82]	RCT	80	SMS programs to increase physical activity
	Willcox et al [37]	RCT	91	Healthy gestational weight gain for obese pregnancies
	Knight et al [19]	One group pilot	10	App with information for lifestyle behavior
	Waring et al [33]	Cross sectional	64	Survey on interest in lifestyle app or website
	Choi et al [36]	RCT pilot	30	Activity app+pedometer wearable
	Lewis et al [83]	Observational cohort	37	Exercise with SMS or app-based support
	Guo et al [84]	One group pilot	50	Video program with yoga via Facebook or DVD
	Heminger et al [42]	Systematic review (with 7 RCTs)	702	SMS or app support on smoking: quitting date, relapse, information, daily messages
**Gestational diabetes mellitus (13 studies)**				
	Ming et al [54]	Systematic review (with 7 RCTs)	579	Telemedicine for diabetes in pregnancy
	Rasekaba et al [53]	Systematic review (with 3 RCTs)	243	Telemedicine for glucose monitoring
	Kruger et al [85]	RCT	18	Telemedicine for glucose monitoring
	Dalfra et al [86]	RCT	276	Telemedicine for glucose monitoring
	Perez-Ferre et al [52]	RCT	100	Telemedicine for glucose monitoring
	Wojcicki et al [87]	RCT	30	Telemedicine for glucose monitoring
	Carral et al [49]	Prospective cohort	104	Web-based telemedicine system
	Given et al [50]	Feasibility study	50	Web-based telemedicine system
	Nicholson et al [88]	Feasibility study	23	Web-based self monitoring, diary
	Mackillop et al [51]	Pilot study	48	Smartphone app with blood glucose meter
	Ganapathy et al [89]	Pilot study	50	Remote blood pressure measurements
	Khorshidi et al [45]	RCT	80	Postpartum screening after GDM^d^
	Harrison et al [90]	Survey+interviews	70	Acceptability of telemedicine for GDM patients

^a^RCT: randomized controlled trial.

^b^SMS: short message services.

^c^BMI: body mass index.

^d^GDM: gestational diabetes mellitus.

**Table 3:** Health outcome of electronic health (eHealth) use in electronic mental (e-mental) health, low- and middle-income countries, and telemonitoring and teleconsultation in pregnancy: overview of the literature.

Study domain and reference		Methods	N	Technology/eHealth intervention
**E-mental health (16 studies)**				
	Lau et al [64]	Systematic review (with 8 RCTs^a^)	1523	Therapist-supported internet-based cognitive behavior therapy among postpartum women
	Lee et al [61]	Systematic review (with 4 RCTs)	1274	Cognitive behavioral therapy with internet
	Ashford et al [63]	Systematic review (with 11 studies)	1537	Web-based perinatal mental health interventions
	Milgrom et al [91]	RCT	43	Cognitive behavioral therapy with internet
	Ngai et al [92]	RCT	397	Telephone-based cognitive-behavioral Therapy
	Shamshiri Milani et al [93]	RCT	54	Telephone-based cognitive-behavioral therapy
	Kingston et al [60]	RCT	636	Acceptability of e-screening for mental health
	Fontein et al [94]	Before-after study	433	Website for maternal stress prevention
	Jimenez-Serrano et al [59]	Prospective cohort	1880	App screening for postpartum depression
	Posmontier et al [62]	Prospective cohort	61	Telephone-administered psychotherapy
	Letourneau et al [65]	Prospective cohort	64	Telephone-based peer support intervention
	Broom et al [95]	Observational	54	Supportive text messaging in postpartum depression
	Mitchell et al [58]	Cross sectional	106	Telephone screening for postpartum depression
	Figueiredo et al [96]	Cross sectional	90	Telephone screening for postpartum depression
	Pugh et al [97]	Case study	1	Therapeutic assistance with email and SMS^b^
	Pineros-Leano et al [98]	Qualitative	25	Screening for postpartum depression using mobile health
**Low and middle income countries (2 studies)**						
	Lee et al [67]; Sondaal et al [66]	2 systematic reviews with 18 RCTs and 18 observational studies	34,149	Mobile health interventions for prenatal, birth, and postnatal period in low- and middle-income countries
**Telemonitoring and teleconsulting (12 studies)**						
	Tapia-Conyer et al [75]	RCT	153	Wireless antepartum maternal-fetal monitoring
	Pflugeisen et al [74]	Non-RCT	1058	Prenatal care with virtual visits and home measurements
	Ivey et al [99]	Prospective cohort	155	Teleconsultation with tertiary center
	Cuneo et al [100]	Prospective cohort	125	Home fetal heart monitoring for anti-SSA+^c^ patients
	Rauf et al [73]	Prospective cohort	70	Fetal monitoring system for induction of labor
	Krishnamurti et al [101]	Prospective cohort	16	Smartphone app with information and symptom scores
	Rhoads et al [102]	Non-RCT	50	Telemonitoring of postpartum hypertension
	Kerner et al [77]	Feasibility study	36	Self-administered fetal heart rate monitoring
	Marko et al [103]	Feasibility study	8	Remote monitored pregnancy care (blood pressure, weight)
	Marko et al [76]	Controlled trial	100	Prenatal care with app and telemonitoring
	Lanssens et al [104]	Retrospective cohort	166	Remote monitoring of hypertension in pregnancy
	Pflugeisen et al [105]	Cross sectional	171	Satisfaction with virtual obstetric care

^a^RCT: randomized controlled trial.

^b^SMS: short message services.

^c^Anti-SSA: Anti-Sjögren’s-syndrome-related antigen A.

#### Mental Health

Electronic mental health has already proven to be successful in general population mental health management [56]. In 16 studies, the applicability on screening for and treatment of postpartum depression was investigated (Table 3). The prevalence of postpartum depression is 3%-15%. These women are reluctant to seek medical attention despite the heavy burden of disease, most notably because of the fear of their child being taken away from them [57,58]. Screening with telephone (alpha coefficients of .72-.94), app (sensitivity 72% and specificity 73%), and iPads were found feasible and acceptable [58-60]. eHealth programs (eg, online sessions based on cognitive behavior therapy) effectuate significant reductions in the depression scales and on symptom scores compared with treatment as usual [61-64]. Besides this significant effect size favoring eHealth, in 1 intervention group, the depression scores reduced also more quickly compared with the waiting list comparator group [63]. Perceptions of peer and social support significantly improved, and higher support was significantly related with lower depression symptoms [65]. An antenatal, first trimester eHealth intervention on depressive symptoms showed 80% intervention response and 60% remission (n=12) [63].

#### Low- and Middle-Income Countries

Limited resources and poor information are still leading to preventable maternal and neonatal deaths in low- and middle-income countries. The availability of mobile phones (in Africa and South-East Asia over 69%-90%) gives rise to the implementation of eHealth interventions and remote care. For more detailed information in this distinct population where eHealth is widely used, we refer to 2 recently published systematic reviews (Table 3). In summary, the interventions did increase antenatal care attendance, facility and service utilization, skilled support at birth, and vaccination rate [66]. Most of the included studies were of poor methodological quality or did not assess health outcomes [67]. Insufficient information was provided to evaluate the impact of eHealth solutions on maternal and fetal outcomes in these countries [67].

#### Telemonitoring and Teleconsulting

Telemonitoring of pregnancy is perceived to be one of the most promising answers to the possibilities of eHealth in pregnancy. Several hardware and software systems involving more complex remote monitoring are described lately (Table 3). An integrated system for maternal monitoring of glucose, weight, pulse and blood pressure, and a chat feature for clinician-patient contact is now in test [68]. Yi et al developed an Android-based mobile terminal for wireless fetal monitoring and uterine contractions tracking [69]. Using this system, patients in rural areas are provided with telemonitoring without traveling or hospitalization. Several other telemonitoring devices for cardiotocography have been tested in pilot settings or prospective cohorts and found feasible [70-72]. Currently, the effects of maternal and fetal telemonitoring in high-risk pregnancies on outcome, satisfaction, and costs are under research compared with hospital admission (the HOTEL trial, registered under #NTR6076). In a pilot with remote monitoring with transabdominal fetal electrocardiography (f-ECG) after induction with dinoprostone pessaries (n=70), successful monitoring was obtained in 89% [73]. Three women were recalled to the hospital due to suspicious f-ECG, of which in 2 cases caesarean section was indicated. A *Virtual Obstetric Care* program with normal visits combined with teleconferencing visits for low-risk pregnancy showed no increased risks in health outcomes besides an increase in preeclampsia diagnosis [74]. Another demonstration project describes a promising system of a wirelessly enabled maternal-fetal monitoring system *MiBebe*, used for the improvement of perinatal care in rural regions in Mexico. In the group of 153 high-risk pregnancies, the remote monitoring in 74 patients resulted in markedly increased adherence to antenatal visits with no adverse health outcomes compared with usual care [75]. One pilot study describes an alternative prenatal care schedule, including an integrated technology platform (mobile app, wireless weight scale, and blood pressure cuff), leading to a 43% reduction in outpatient visits (8 vs 14 visits) [76]. There was an increase in satisfaction and patient engagement and no change in perinatal outcome despite the decrease in face-to-face contact [76]. Remote monitoring and consultation can potentially reduce outpatient visits for antenatal consultation as well as hospitalization for certain clinical reasons. We see this in managing gestational diabetes with glucose monitoring but also in fetal monitoring for fetal growth restriction [53,77]. A model of cost-effectiveness analysis in a tertiary hospital (Ghent, Belgium) predicted a cost-reduction of 145,822 euros per year achieved by introducing home monitoring in high-risk pregnancy [78].

### Patient and Caregiver Experience

Examining patients’ satisfaction with eHealth interventions, users describe high convenience and acceptance resulting in more patient activation and education. Patients report less concerns and anxiety and are comfortable with fewer clinic visits. Satisfaction rates vary between 86% and 95% in e-mental health studies and 90% (46/51) in home-monitored induction patients, who were very glad to stay in their own homely ambience as long as possible [73,79].

On the health care providers’ point of view, adaptation of obstetricians and midwives to eHealth solutions has not been widely described. Only 1 qualitative study interviewed 12 health care providers in obstetric departments. Concerns were raised on implementation barriers and potential medico-legal risks, but if addressed properly, implementation was considered feasible. Some clinicians admitted to have insufficient familiarity and skill with eHealth limiting their engagement and comprehension of the possibilities that eHealth technologies can confer to perinatal care. Overall, these clinicians regarded telemedicine as an additional parallel service rather than integrated into the antenatal care model [27].

## Discussion

### Principal Findings

By providing this overview of the literature, we aimed to assess the applicability, advantages, and limitations of the use of eHealth in perinatal care. This review showed that eHealth interventions have a very broad, multilevel field of application focused on perinatal care in all its aspects. Most of the reviewed 71 articles were published after 2013, suggesting this novel type of care is an important topic of clinical and scientific relevance. Women of reproductive age seem to be interested in eHealth, as shown by their frequent use of smartphone, internet and apps, and searches for pregnancy information. Most health outcomes for perinatal eHealth interventions were generally positive, either resulting in positive effects (lifestyle, mental health) or providing multiple advantages while health outcomes were found equal (diabetes care). The implementation of telemonitoring was not studied extensively, but research provided important effects and advantages on facilitation of new care models. Patient and care provider satisfaction with eHealth interventions rates are generally good, with rates up to 95%.

### Additional Considerations

Despite the promising preliminary results as reviewed above, research in eHealth has progressed much slower than developments in the health technology industry. A great amount of the reviewed articles on this subject addressed more than health outcomes or satisfaction rates alone. Advances in (implementation of) apps and devices and patient-generated data are retained by legal and financial concerns. Possible privacy risks involve a lack of control to collection of data and the use by third parties afterwards.

In the United States, eHealth legislation, secured in the Fair Information Practice principles (part of the Health Insurance Portability and Accountability Act), is lacking protection for endpoint users: the patients. End-to-end data encryption can be used to protect the useful patient data. Combined with authentication and access control mechanisms for patients as well as care providers, eHealth technologies can further enhance final security control [106]. The development of the Telemedicine for Medicare Act of 2015 may accelerate the removal of barriers and limitations regarding use of telehealth between different states in the United States [107].

In the framework of European law, eHealth is simultaneously a health care service and an information service with corresponding legislation [108]. eHealth developers have to mind general legislation regarding privacy protection (Dir 95/46/EC, Arts 8-12), electronic identification services, e-Commerce directive (eg, online contracting), safety requirements of medical devices, and general product safety and liability requirements. In answer to the interstate developments in eHealth care, the Cross Border Directive was initiated in 2011 in the European Union (EU). The objective of the initiatives within this directive is to turn telemedicine into a standard medical service, accessible to every European patient and fully covered by the respective social security system. Difficulties arise on liability and creating uniform rules in the EU, as member states have very intrinsic differences in national rules on health care, privacy, and liability. One advice would be for each member state to provide a legal framework for telemedicine, whereas the role of the EU would be limited to regulation [108].

The costs associated with development, purchase, and maintenance of eHealth equipment have dropped in recent years due to technological advancements [107]. Primary investments to implement eHealth in perinatal care are now attributed to personnel costs for both providers and technical support. However, to deliver care with the help of eHealth can also create savings on personnel costs and clinic visits. A systematic review of economic evaluation in telehealth solutions concluded that 29 out of 39 studies (74%) reported cost-effective, economically beneficial eHealth interventions in different conditions and diseases. The conclusion highlighted the fact that many studies did not report all recommended economic outcome items, leading to inconsistent analyses [109].

The challenges for reimbursement are delaying the widespread adoption of eHealth in all ranges of sections of hospital care. Coverage is fragmented, varying at level of country, within hospitals in the same country and within different specialties of health care [29]. Health insurance companies seem to be inclined to cover only well-researched eHealth interventions with according economic evaluations. The use of low-risk, inexpensive care models can operate as opportunities to objectify possible reduction in health care costs.

**Advantages and disadvantages of eHealth implementation in perinatal care.**

Advantages

- Patient satisfaction
- Patient engagement
- Fewer clinic visits
- Clinician satisfaction
- Remote monitoring
- Access to care in low- and middle-income countries

Disadvantages

- Reimbursement
- Legal issues
- Technical issues

Indistinct

- Impact on health outcome
- Impact on costs
- Limited A-level evidence

Successes will motivate policy makers and drive the insurance market for additional coverage. Rigorous medical evidence can act as an extra stimulant; however, the duration and costs of designs and trials need to be taken into consideration [107].

### Conclusion and Future Perspectives

This review provided an overview of eHealth as the next-generation perinatal care. Textbox 1 provides a condensed summary of the advantages (as described in Principal Findings) and disadvantages (as described in Additional Considerations) of the implementation of eHealth in perinatal care. If eHealth is to achieve its full potential, it should attain all domains of quality in care including safety, timeliness, effectiveness, efficiency, and patient centeredness. Cost-effectiveness assessment is needed to rationalize embracement and reimbursement. Policy makers should consider the international frameworks of legislation to support and implement this new form of care.

We accentuate that more research is needed, including economic evaluation of eHealth interventions. Growing engagement of calls for funding have responded: more large funding associations focus on the use of eHealth, warranting the qualitative impact of the studies in the application designs [110]. In addition, the potential of technology raised a nearly quadrupled amount of money in venture capital funding, from US $1.1 billion in 2011 to US $4.3 billion in 2015 [111].

Despite the challenges of privacy, liability, and costs, eHealth is very likely to disperse globally in the next decade. Some even state health care is approaching a tipping point [112]. The current shift to patient-centered care and increased patient empowerment underlines the need for revising current medical practice. eHealth has the potential to be integrated into standard care and deliver a revolution in perinatal health.

## Appendix

Multimedia Appendix 1 Search strategy and flow diagram.

## Abbreviations

Anti-SSA: Anti-Sjögren’s-syndrome-related antigen A
eHealth: electronic health
e-mental health: electronic mental health
EU: European Union
f-ECG: fetal electrocardiography
GDM: gestational diabetes mellitus
RCT: randomized controlled trial
SMS: short message service
