# Supplementary material for: eHealth as the Next-Generation Perinatal Care: An Overview of the Literature
Source: J Med Internet Res. 2018 Jun 5;20(6):e202. doi: 10.2196/jmir.9262 (PMC6008510; doi:10.2196/jmir.9262)

Multimedia appendix 1: Search strategy and flow diagram

1. Pubmed

((#1) OR (#2)) AND ((#3) OR (#4)) OR (#5)

(1) (pregnancy[Title/Abstract] OR mother*[Title/Abstract] OR women, pregnant[MeSH Terms] OR woman, pregnant[MeSH Terms])

(2) ((((((Fetus[MeSH Terms]) OR Fetus[Title/Abstract]) OR Fetuses[Title/Abstract]) OR Fetal Structures[Title/Abstract]) OR Fetal Structure[Title/Abstract]) OR Structure, Fetal[Title/Abstract]) OR Structures, Fetal[Title/Abstract]

(3) (mhealth[Title/Abstract] OR m-health[Title/Abstract] OR mobile health[Title/Abstract] OR telemedicine[Title/Abstract] OR telehealth[Title/Abstract] OR telecare[Title/Abstract] OR telehealth care[Title/Abstract] OR mobile telehealth care[Title/Abstract] OR mobile telemedicine[Title/Abstract] OR mcare[Title/Abstract] OR m-care[Title/Abstract] OR mobile communication[Title/Abstract] OR mobile technolog*[Title/Abstract] OR multimedia technolog*[Title/Abstract] OR mobile devic*[Title/Abstract] OR app[Title/Abstract] OR apps[Title/Abstract] OR mobile app*[Title/Abstract] OR portable electronic app*[Title/Abstract] OR portable software app*[Title/Abstract] OR voice messag*[Title/Abstract] OR website*[Title/Abstract] OR radio[Title/Abstract] OR cell phone*[Title/Abstract] OR cellular phone*[Title/Abstract] OR telephone*[Title/Abstract] OR cellular telephone*[Title/Abstract] OR portable cellular phone*[Title/Abstract] OR transportable cellular phone*[Title/Abstract] OR mobile phone*[Title/Abstract] OR smart phone*[Title/Abstract] OR smart-phone*[Title/Abstract] OR smartphone*[Title/Abstract] OR mobile tablet computer*[Title/Abstract] OR tablet computer*[Title/Abstract] OR

handheld computer*[Title/Abstract] OR microcomputer*[Title/Abstract] OR personal digital assistant*[Title/Abstract] OR PDA[Title/Abstract] OR text messag*[Title/Abstract] OR SMS[Title/Abstract] OR short messag*[Title/Abstract] OR multimedia messag*[Title/Abstract] OR multi-media messag*[Title/Abstract] OR podcast*[Title/Abstract] OR mp3[Title/Abstract] OR social media[Title/Abstract] OR social network*[Title/Abstract] OR facebook[Title/Abstract] OR twitter[Title/Abstract] OR twitter messag*[Title/Abstract] OR ehealth[Title/Abstract] OR e-health[Title/Abstract] OR telemedicine[MeSH Terms] OR short message service[MeSH Terms] OR cellular phone[MeSH Terms] OR mobile applications[MeSH Terms] OR text messaging[MeSH Terms] OR radio[MeSH Terms] OR computers, handheld[MeSH Terms] OR multimedia[MeSH Terms] OR social media[MeSH Terms] OR internet[MeSH Terms])

(4) (((((Fetal monitoring[MeSH Terms]) OR Fetal monitoring[Title/Abstract]) OR Monitoring, Fetal[Title/Abstract]) OR Fetal Monitorings[Title/Abstract]) OR Monitorings, Fetal[Title/Abstract]

(5) (Remote[Title/Abstract] OR self-administered[Title/Abstract] OR home[Title/Abstract] OR (home based) [Title/Abstract] OR phonocardiograph[Title/Abstract])

2. Embase

((#1) OR (#2)) AND ((#3) OR (#4)) OR (#5)

(1) Pregnancy/exp OR ‘child bearing':ab,ti OR ‘childbearing':ab,ti OR ‘gestation':ab,ti OR ‘gravidity':ab,ti OR ‘intrauterine pregnancy':ab,ti OR ‘pregnancy maintenance':ab,ti OR ‘pregnancy trimesters':ab,ti

(2) Fetus/exp OR ‘fetal period':ab,ti OR ‘fetal phase':ab,ti OR ‘fetal stage':ab,ti OR ‘foetal period':ab,ti OR ‘foetal phase':ab,ti OR ‘foetal stage':ab,ti OR ‘foetus':ab,ti

(3) (mhealth:ab,ti OR 'm health':ab,ti OR 'mobile health':ab,ti OR ehealth:ab,ti OR 'e health':ab,ti OR telemedicine:ab,ti OR telehealth:ab,ti OR telecare:ab,ti OR 'telehealth care':ab,ti OR 'mobile telehealth care':ab,ti OR 'mobile telemedicine':ab,ti OR mcare:ab,ti OR 'm care':ab,ti OR 'mobile communication':ab,ti OR 'mobile technology':ab,ti OR 'mobile technologies':ab,ti OR 'multimedia technology':ab,ti OR 'multimedia technologies':ab,ti OR 'mobile device':ab,ti OR 'mobile devices':ab,ti OR 'app':ab,ti OR 'apps':ab,ti OR 'mobile application':ab,ti OR 'mobile applications':ab,ti OR 'mobile app':ab,ti OR 'mobile apps':ab,ti OR 'portable electronic app':ab,ti OR 'portable electronic apps':ab,ti OR 'portable electronic application':ab,ti OR 'portable electronic applications':ab,ti OR 'portable software app':ab,ti OR 'portable software apps':ab,ti OR 'portable software application':ab,ti OR 'portable software applications':ab,ti OR 'voice message':ab,ti OR 'voice messages':ab,ti OR radio:ab,ti OR 'cell phone':ab,ti OR 'cell phones':ab,ti OR 'cellular phone':ab,ti OR 'cellular phones':ab,ti OR telephone*:ab,ti OR 'cellular telephone':ab,ti OR 'cellular telephones':ab,ti OR 'portable cellular phone':ab,ti OR 'portable cellular phones':ab,ti OR 'transportable cellular phone':ab,ti OR 'transportable cellular phones':ab,ti OR 'mobile phone':ab,ti OR 'mobile phones':ab,ti OR 'smart phone':ab,ti OR 'smart phones':ab,ti OR smartphone*:ab,ti OR 'smart-phone':ab,ti OR 'smart-phones':ab,ti OR 'mobile tablet computer':ab,ti OR 'mobile tablet computers':ab,ti OR 'tablet computer':ab,ti OR 'tablet computers':ab,ti OR 'handheld computer':ab,ti OR 'handheld computers':ab,ti OR microcomputer*:ab,ti OR 'personal digital assistant':ab,ti OR 'personal digital assistants':ab,ti OR pda:ab,ti OR 'text message':ab,ti OR 'text messages':ab,ti OR sms:ab,ti OR 'short message service':ab,ti OR 'short message services':ab,ti OR 'multimedia message':ab,ti OR 'multimedia

messages':ab,ti OR 'multi-media message':ab,ti OR 'multi-media messages':ab,ti OR podcast*:ab,ti OR mp3:ab,ti OR 'social media':ab,ti OR 'social network':ab,ti OR 'social networks':ab,ti OR facebook:ab,ti OR twitter:ab,ti OR 'twitter message':ab,ti OR 'twitter messages':ab,ti OR 'telemedicine'/exp OR 'telemedicine')

(4) (Fetus monitoring:/exp, ‘electronic fetal monitoring':ab,ti OR ‘electronic foetal monitoring':ab,ti OR ‘fetal monitoring':ab,ti OR ‘foetal monitoring':ab,ti OR ‘foetus monitoring':ab,ti OR ‘intrauterine monitoring':ab,ti OR ‘monitoring, fetus':ab,ti OR ‘uterine monitoring':ab,ti

(5) (‘Remote':ab,ti OR ‘self-administered':ab,ti OR ‘home':ab,ti OR ‘home based':ab,ti OR ‘phonocardiograph':ab,ti)

Figure M1. Flow diagram of abstract and article review of literature search

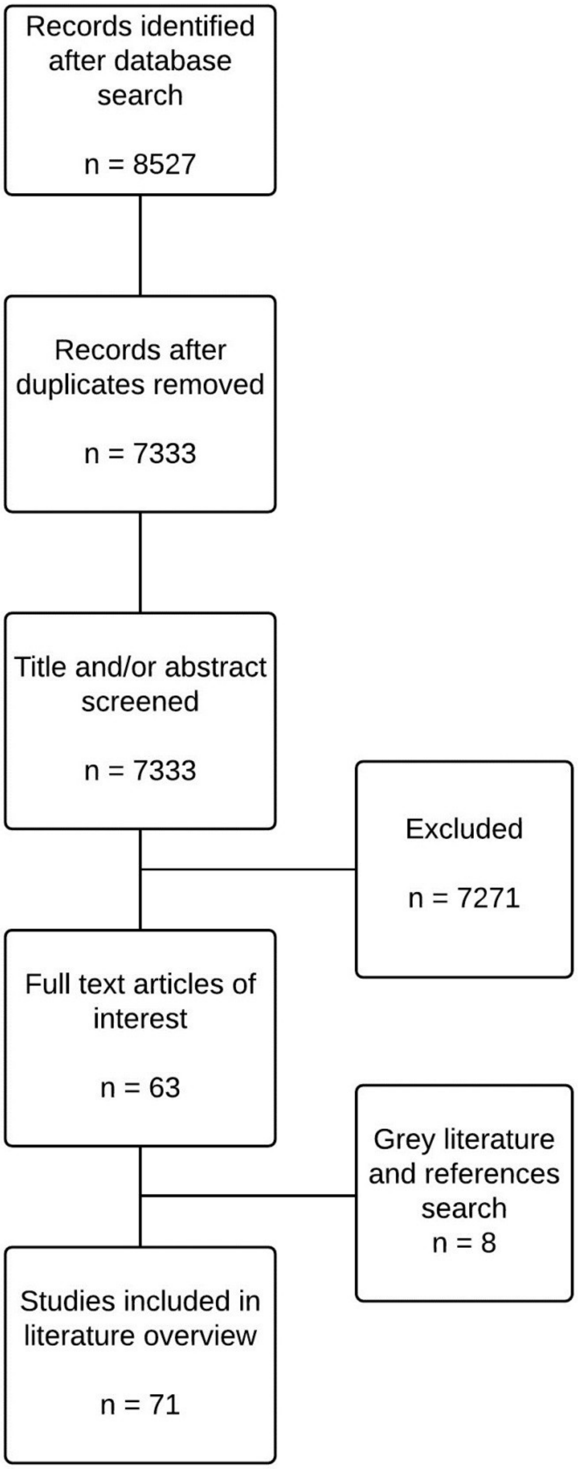

Supplement: Multimedia Appendix 1 [file jmir_v20i6e202_app1.pdf]
